# Growth Enhancement of Probiotic *Pediococcus acidilactici* by Extractive Fermentation of Lactic Acid Exploiting Anion-Exchange Resin

**DOI:** 10.3389/fmicb.2018.02554

**Published:** 2018-10-29

**Authors:** Majdiah Othman, Arbakariya B. Ariff, Mohd Rizal Kapri, Leonardo Rios-Solis, Murni Halim

**Affiliations:** ^1^Department of Bioprocess Technology, Faculty of Biotechnology and Biomolecular Sciences, Universiti Putra Malaysia, Serdang, Malaysia; ^2^Bioprocessing and Biomanufacturing Research Center, Faculty of Biotechnology and Biomolecular Sciences, Universiti Putra Malaysia, Serdang, Malaysia; ^3^School of Engineering, Institute for Bioengineering, University of Edinburgh, Edinburgh, United Kingdom

**Keywords:** probiotic, lactic acid, anion exchange resin, Amberlite IRA 67, extractive fermentation, end-product inhibition

## Abstract

Fermentation employing lactic acid bacteria (LAB) often suffers end-product inhibition which reduces the cell growth rate and the production of metabolite. The utility of adsorbent resins for *in situ* lactic acid removal to enhance the cultivation performance of probiotic, *Pediococcus acidilactici* was studied. Weak base anion-exchange resin, Amberlite IRA 67 gave the highest maximum uptake capacity of lactic acid based on Langmuir adsorption isotherm (0.996 g lactic acid/g wet resin) compared to the other tested anion-exchange resins (Amberlite IRA 410, Amberlite IRA 400, Duolite A7 and Bowex MSA). The application of Amberlite IRA 67 improved the growth of *P. acidilactici* about 67 times compared to the control fermentation without resin addition. Nevertheless, the *in situ* addition of dispersed resin in the culture created shear stress by resins collision and caused direct shear force to the cells. The growth of *P. acidilactici* in the integrated bioreactor-internal column system containing anion-exchange resin was further improved by 1.4 times over that obtained in the bioreactor containing dispersed resin. The improvement of the *P. acidilactici* growth indicated that extractive fermentation using solid phase is an effective approach for reducing by-product inhibition and increasing product titer.

## Introduction

High viable cell density in cultivation of LAB is vital to get the valuable biomass to be profitably applied as a probiotic ingredient in various products and proteins sources for human and animal consumptions (Hayek and Ibrahim, 2013; Halim et al., 2017). In the meantime, lactic acid produced from the fermentation of LAB has numerous applications such as in food, chemical, pharmaceutical, and cosmetic industries. The worldwide demand of lactic acid is estimated to be approximately 130,000 to 150,000 tons per year (Randhawa et al., 2012) and it is also expected that the globally usage of lactic acid will increase rapidly in the near future (Wee et al., 2006). These aspects influence the growing interest for more LAB related research and development from researchers and industries (Hayek and Ibrahim, 2013).

The presence of inhibitors such as substrate and product inhibitions that inhibit the cell growth and reduce the product yield is one of the main problems in fermentation process (Yuwono et al., 2008). The inhibition exerted by lactic acid in LAB fermentation can either be competitive or non-competitive inhibition. In comparison, the effect of lactic acid inhibitory on the cell growth was shown to be stronger than the effect on fermentation activity (Milcent and Carrere, 2001). The inhibitory effect of lactic acid on cell metabolism and proliferation might be due to the increment in medium osmotic pressure (Cui et al., 2016) and other fermentation by-products such as sodium formate, acetic acid, and formic acid that causes an individual inhibitory effect (Loubière et al., 1997). Furthermore, fermentation of LAB often suffers end-product inhibition due to the acidification of cytoplasm and failure of proton motive forces (Wee et al., 2006). The acidification of cytoplasm is caused by the undissociated lactic acid that passes through the bacterial membrane and dissociates inside the cell. The transmembrane pH gradient is affected by the undissociated lactic acid that is soluble and the dissociated lactate which is insoluble within the cytoplasmic membrane. Eventually, this reduce the amount of energy available for cell growth. Therefore, to reduce the inhibitory effect of lactic acid during fermentation process, lactic acid must be removed selectively *in situ* from the culture (Othman et al., 2017a).

Lactic acid bacteria fermentations are characterized by the kinetic of product inhibition that affects growth rate and productivity (Luedeking and Piret, 2000). Various fermentation strategies have been developed to overcome the product inhibition such as neutralization of the acid formed (Juturu and Wu, 2016), solvent extraction (Chen et al., 2012), electrodialysis (Habova et al., 2004) and application of fed batch fermentation (Ming et al., 2016; Othman et al., 2017b). Lactic acid extractive fermentation using anion exchange resin to reduce product inhibition due to lactic acid accumulation in the fermentation of LAB has also been reported (Garret et al., 2015; Cui et al., 2016). In general, the successful application of resin as lactic acid adsorbent may be influenced by several factors including the selection of resin, biocompatibility with microorganisms and resin regenerability. There are a few criteria that can be used for resin selection such as a strong ability in adsorbing lactic acid and high specificity in adsorbing lactic acid to avoid adsorbing other substrates and products such as glucose and amino acids that are also present in the system (Gao et al., 2010). The biocompatibility of resin with microorganism is also important to avoid toxicity effects that may decrease the viability of cells during the process (Pradhan et al., 2017). Regenerability of resins allows reusability after regeneration process according to the manufacturer’s instruction (Cui et al., 2016). Efficient regenerability of resins may increase resin service life and in turn reduced the processing cost. Resins are considered solid waste and hence care must be taken to dispose of the resin in compliance with waste management regulations. To date, not much literature is available on the mechanism of lactic acid removal using anion exchange resins and its effect on growth of LAB. Instead, most of the reported studies are focusing on the strategy for solely improving lactic acid production instead of viability of cell biomass that are important in probiotic industry (Boonmee et al., 2016; Nguyen et al., 2016).

The purpose of this study was to investigate the possibility of using anion exchange resins for *in situ* lactic acid removal in cultivation of *P. acidilactici* for growth enhancement. Different types of anion-exchange resins were tested and the effect of loading concentrations of the selected resin in the culture was studied. The anion exchange resin stability and efficiency in removing lactic acid and enhancing growth of *P. acidilactici* were studied by conducting fermentation with the application of anion exchange resin at different agitation speed and different types of bioreactor systems, with and without an integrated bioreactor-internal column system. The results of this study may provide an alternative to overcome product inhibition in LAB fermentation.

## Materials and Methods

### Microorganism, Culture Maintenance, and Inoculum Preparation

*P. acidilactici* DSM 20238 (was purchased from DSMZ-German Collection of Microorganisms and Cell Cultures) was grown and maintained in De Man, Rogosa and Sharpe (MRS) medium (Cat. no. 110661 from Merck Millipore, Germany). Glycerol stock of *P. acidilactici* was prepared by inoculating the strain [10% (vv^-1^)] into 250 mL Erlenmeyer flask containing 50 mL MRS medium. The flask was incubated in an incubator shaker (Certomat^®^ BS-1 Braun, Germany) at 37°C, 200 rpm for 24 h. The cells were harvested by centrifugation (Eppendorf Centrifuge 5810 R) at 10,000 rpm for 10 min. The cell pellets obtained were resuspended in 15% (vv^-1^) autoclaved sterile glycerol solution. The glycerol stock of *P. acidilactici* was maintained at -20°C throughout the study. For inoculum preparation, a single colony of *P. acidilactici* was picked up from MRS agar plate and inoculated into 50 mL MRS medium and incubated in an orbital shaker at 37°C and 200 rpm for 10 h.

### Anion-Exchange Resins and Lactic Acid Adsorption Capacity

Five anion-exchange resins (Amberlite IRA 400 CI, Cat. no. 247669, Amberlite IRA 410 CI, Cat. no. 216569, Dowex Marathon MSA, Cat. no. 428760, Amberlite IRA 67, Cat. no. 476633 and Duolite A7, Cat. no. 436704) were purchased from Sigma, Germany. All the resins were sterilized using ultraviolet radiation by exposure to UV lamp in a laminar flow cabinet (for 1 h at room temperature) before used in the experiments. For sorption equilibrium studies, each resin was separately added into 15 mL falcon tube containing 10 mL lactic acid at concentrations between 2 and 15 gL^-1^ in MRS medium. The tubes were agitated on shaker until the sorption reached equilibrium at 200 rpm for 24 h. Resins were then separated from the solution by centrifugation at 10,000 rpm for 10 min. The supernatants were used for determination of remaining lactic acid left in the solution. Resins can be reused by washing with distilled water followed by regenerating with 4% NaOH to elute the lactic acid adsorbed on the resin at an ambient temperature. The wet resin was allowed to dry, and UV sterilized by exposure to UV lamp in a laminar flow cabinet (for 1–2 h at room temperature). The specific uptake capacity of lactic acid (q) for the Langmuir isotherm model was determined by using Equation 1:

(1) $$q=\frac{\left(C_{i}-C_{\mathrm{eq}}\right)}{X}$$

From Equation 1, *q* (g of lactic acid/g of biosorbent) is the specific uptake capacity of lactic acid, *C*_i_ (gL^-1^) is the initial concentration of lactic acid, *C*_eq_ (gL^-1^) is the equilibrium concentration of lactic acid and *X* (gL^-1^) is the concentration of biosorbent in solution. The Langmuir sorption model as described in Equation 2 was selected for the fitting of experimental data:

(2) $$\frac{1}{q_{e}}=\left(\frac{1}{K_{a}q_{m}}\right)\frac{1}{C_{e}}+\frac{1}{q_{m}}$$

From Equation 2, *q*_m_ (g lactic acid/g wet resin) is the maximum uptake capacity of lactic acid, *q*_e_ is the specific uptake capacity of lactic acid, *C*_e_ is the equilibrium concentration of lactic acid and *K*_a_ (Lg^-1^) is the equilibrium constant of the Langmuir isotherm model.

### Cultivation of *P. acidilactici* and Experimental Design

For preliminary experiments of studying the effect of *in situ* addition of different types of resins, fermentations were performed in 500 mL Erlenmeyer flasks containing 100 mL MRS medium and 10 gL^-1^ of anion-exchange resin. Following the result obtained from the experiments, different loadings of Amberlite IRA 67 (0–40 gL^-1^) were added into the culture to further study the effect of resin loadings on the performance of batch fermentation of *P. acidilactici.* The anion-exchange resins were sterilized using ultraviolet radiation (as described in the previous section) prior addition into the flasks during inoculation. The flasks were inoculated with 10% (vv^-1^) inoculum and incubated at 37°C, 200 rpm for 24 h without pH control.

**FIGURE 1:**
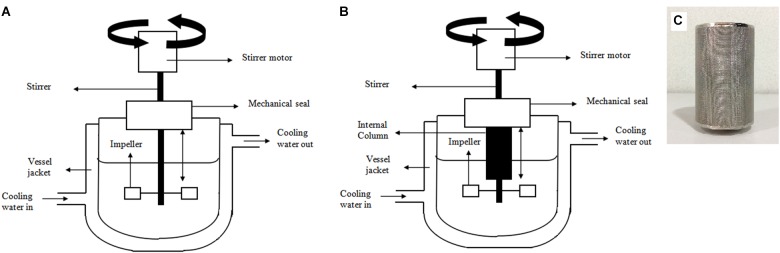
Schematic diagrams of **(A)** 2 L stirred tank bioreactor **(B)** 2 L stirred tank bioreactor with integrated internal column system **(C)** internal column applied in the 2 L stirred tank bioreactor with integrated internal column system.

Batch cultivation of *P. acidilactici* was carried out in a 2 L stirred tank bioreactor (STR) (BIOSTAT, B. Braun Biotech International, GmbH, Germany) (Figure 1A). The STR was equipped with a thermostat jacket system, a six blade Rushton turbine and control systems for dissolved oxygen, temperature and pH. The STR was initially filled with 1 L of MRS medium and the reactor was set to be carried out under facultative condition, in which the broth was sparged with oxygen gas at 0.1 vvm (0.15 L/min) until the dissolved oxygen level reached 100%. The oxygen supply was then stopped, and 10% (vv^-1^) *P. acidilactici* inoculum was aseptically inoculated into the STR. The batch fermentation was preceded without aeration, agitated at 300 rpm, 37°C. The pH was not controlled but the changes were monitored throughout the fermentation. The effect of agitation speed on the stability of Amberlite IRA 67 (10 gL^-1^) and cultivation performance of *P. acidilactici* was conducted at agitation speeds of 200 rpm, 300 rpm, and 400 rpm.

Fermentation of *P. acidilactici* was also performed with an integrated bioreactor-internal column system for simultaneous removal of lactic acid during cultivation (Figure 1B). The column (Figure 1C) was internally attached to the bioreactor and was aseptically packed with UV sterilized Amberlite IRA 67 (10 gL^-1^) during inoculation.

### Analytical Methods

For analysis, 5 mL of culture samples were withdrawn at time intervals. Cell growth of *P. acidilactici* was determined using CFU as described by Ming et al. (2016).

The supernatants obtained from the centrifuged fermentation broths (10,000 rpm for 10 min) were analyzed by RP-HPLC (Waters 2695, Separations Module and Waters 2410, Refractive Index Detector) for determination of glucose and lactic acid profiles. The analysis was conducted using a shodex SH-1011 column (7 μm, 8 mm × 300 mm) that was connected to shodex SH-G guard column (7 μm, 6 × 50 mm). 5 mM sulfuric acid was used as the mobile phase solvent and the system was maintained at 60°C with flow rate of 1.0 mL min^-1^. Empower software was employed for RP-HPLC data processing.

The samples of anion-exchange resins were examined under scanning electron microscope (SEM) (Hitachi S-3400N, Germany). Briefly, the cells fixation was done with 4% (vv^-1^) glutaraldehyde buffer for 12 h at 4°C. The fixed cells were then washed three times with 0.1 M sodium cacodylate buffer for 10 min. After post-fixation of the cells with 1% (wv^-1^) osmium tetroxide for 2 h at 4°C, washing step was repeated once again prior dehydration with increasing serial concentrations of acetone. Samples were carefully mounted on an aluminum stub using a double stick carbon tape and introduced into the chamber of the sputter coater. The samples were then coated with a very thin film of metal gold/palladium (40–60 nm) and viewed in the SEM at 1,000× and 5,000× magnifications.

The lactic acid desorption was done by washing the resin with distilled water followed by regenerating with 4% NaOH to elute the adsorbed lactic acid from the resin at an ambient temperature. Equation 3 can be used to calculate the total amount of lactic acid produced from the fermentation:

(3) $$A(gL^{-1})=B(gL^{-1})+C(gL^{-1})$$

Where, A is total lactic acid produced, B is equilibrium lactic acid in the culture, and C is lactic acid eluted from resin.

### Statistical Analysis

The data was statistically analyzed by SPSS software Version 21 and Microsoft Excel 2010. Results presented are the average of at least three independent replicates and were represented as mean value ± standard deviation. Unpaired *T*-test and one-way analysis of variance (one-way ANOVA) were used to determine the significance differences (*P* < 0.05) between different samples.

## Results and Discussion

### Characteristics and Adsorption Capacity of Anion-Exchange Resins Toward Lactic Acid Selectivity

Three strong base anion resins (Amberlite IRA 400, Amberlite IRA 410 and Dowex Marathon) and 2-weak base anion resins (Amberlite IRA 67 and Duolite A7) were screened for the highest uptake capacity of lactic acid based on adsorption isotherm. The anion-exchange resins were selected based on their type of supporter matrix and exchange capacity for organic acids (Tan et al., 2011).

**FIGURE 2:**
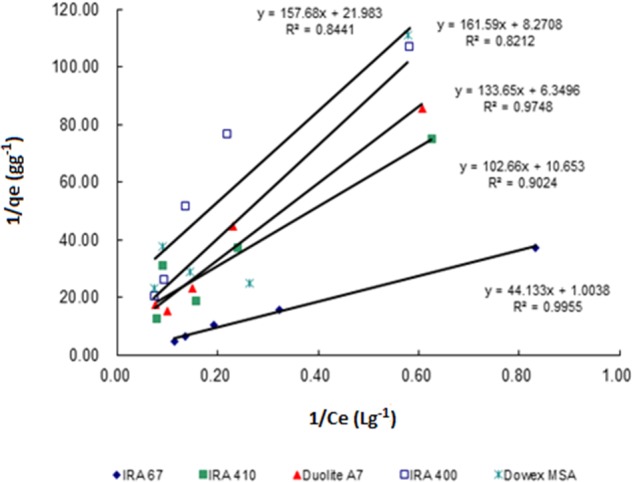
Langmuir biosorption isotherm profile for the uptake of lactic acid by different types of anion exchange resin (30 gL^-1^) in different concentrations of lactic acid (2–15 gL^-1^).

Sorption isotherms described the equilibrium relationship between adsorbent and adsorbate that measure the capacity of an adsorbent for an adsorbate (Ho, 2006). In many cases, acid sorption and equilibrium are interpreted in terms of simple Langmuir type isotherm (Bhandari et al., 2000). The data for the adsorption of lactic acid by all tested resins follows Langmuir adsorption model which describes the pattern of monolayer adsorption of lactic acid onto a homogeneous resin surface (Figure 2). The linear form of the Langmuir isotherm equation (Equation 2) can be used to calculate the maximum uptake capacity of lactic acid (*q*_m_) and sorption equilibrium constant of lactic acid by resins (*K*_a_). Amberlite IRA 67 gave the highest maximum uptake capacity of lactic acid (0.996 g lactic acid/g wet resin) with the highest correlation coefficient (*R*^2^) (0.9955) compared to other anion-exchange resins (Table 1). The regenerated resins that were reused for three consecutive batches showed a similar data (data not shown).

**Table 1:** Characteristic data for Langmuir isotherm and correlation coefficient (*R*^2^) for lactate adsorption by anion-exchange resins at different initial lactate concentrations.

Type of resin	*q*_m_ (gg^-1^)	*K*_a_ (Lg^-1^)	Correlation coefficient (*R*^2^)
IRA 67	0.996	0.023	0.9955
IRA 410	0.094	0.104	0.9024
Duolite A7	0.157	0.048	0.9748
IRA 400	0.045	0.141	0.8212
Dowex MSA	0.121	0.051	0.8441


The data for the adsorption of lactic acid by all selected resins provides information that the weak base resins are more efficient than the strong base resins for lactic acid adsorption. This finding is in an agreement with the previous literature and supported by resin product data sheets stating that weak base resins are preferred than strong base resins for organic acids separation (Bishai et al., 2015). This is mainly owing to the attribute of weak base resins of having higher resistance to oxidation and organic fouling (Gluszcz et al., 2004). Amberlite IRA 67 is based on a matrix of cross linked acrylic gel which is more hydrophilic than styrenic resins (Arup, 1995). The resin selectivity to most of organic acids is higher due to high numbers of aromatic rings in styrenic resins making it hydrophobic.

### Effects of Anion-Exchange Resins on Growth of *P. acidilactici* and Lactic Acid Accumulation

The effect of using different types of resins on growth of *P. acidilactici* and accumulation of lactic acid is shown in Table 2. Maximum viable cell concentration and lactic acid accumulated obtained from the culture with Amberlite IRA 67 were significantly different (*P* < 0.05) compared to the culture without resin and cultures with other resins. Amberlite IRA 67 with 10 gL^-1^ of resin loading gave the highest viable cell concentration (1.3 × 10^11^ CFUmL^-1^) and the lowest lactic acid accumulated (4.74 gL^-1^) compared to the control experiment (no resin) and other tested resins. The observation is in accordance with the biosorption isotherm profile for the uptake of lactic acid by each resin. Anion-exchange resins usually have different levels of affinity toward nutrients and compounds available in the culture which could be the main reason for different inhibitory effect toward cells for every different type of anion-exchange resins (Tan et al., 2011; Halim et al., 2014; Oslan et al., 2017). Thus, Amberlite IRA 67 was selected and used for subsequent experiments to further explore its potential in extractive fermentation of lactic acid.

**Table 2:** Effect of *in situ* addition of different types of anion exchange resins (10 gL^-1^) on the performance of *P. acidilactici* in batch fermentation.

Type of resin	Maximum viable cell concentration (cfu mL^-1^)	Lactic acid accumulated (gL^-1^)
IRA 67	1.3 × 10^11^± 0.04^a^	4.74 ± 0.03^e^
IRA 410	1.0 × 10^11^± 0.07^c^	5.63 ± 0.12^c^
Duolite A7	1.1 × 10^11^ ± 0.06^b^	5.41 ± 0.03^d^
IRA 400	1.0 × 10^11^± 0.11^c^	5.58 ± 0.07^c^
Dowex MSA	1.4 × 10^10^ ± 0.07^d^	5.92 ± 0.06^b^
Control	1.1 × 10^10^± 0.04^e^	7.64 ± 0.02^a^


*The results of maximum viable cell concentration and concentration of lactic acid accumulated are the average of triplicate experiments. a, b, c, d, and e: Mean values with the different letters are significantly different. Statistically significant coefficient (*P* < 0.05) are expressed as means ± SE.*

**FIGURE 3:**
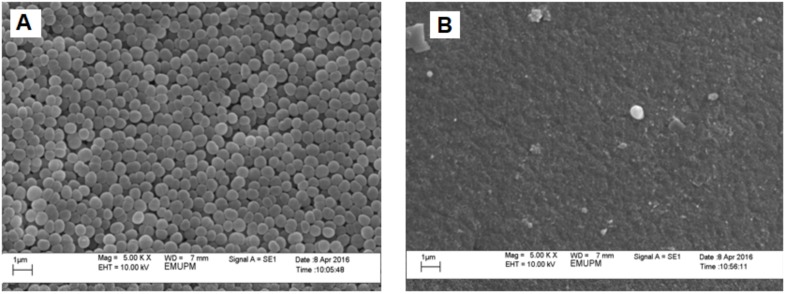
Scanning electron photographs (magnification at ×5,000) of **(A)** control *P. acidilactici* **(B)** IRA 67 resin surface.

The growth morphology of *P. acidilactici* when being cultivated with *in situ* Amberlite IRA 67 addition (Figure 3A) remained similar to the free cells without resin addition (Figure 3B), which was in round shape. Based on the samples viewed under SEM, although some of the cells were shown to be attached on resins surface, however, there was no cells agglomeration observed on the resins surface (Figure 3B). This finding was similar to the study reported by Tan et al. (2011) in which the growth morphology of *E. coli* in fermentation with *in situ* addition of two types of resin (WA30 and M43) was similar to the free cells without resin addition and no cells agglomeration was found on the resins surface although there were some of the cells attached on the resins surface.

### Effect of Different Loadings of Amberlite IRA 67 on Growth of *P. acidilactici* and Lactic Acid Accumulation

Different loadings of Amberlite IRA 67 resin, ranging from 5 to 40 gL^-1^, were used to evaluate the effect of loading concentrations on the performance of batch fermentation of *P. acidilactici.* The best loading concentration of Amberlite IRA 67 resin was found to be 10 gL^-1^ with the highest maximum viable cell concentration of 1.3 × 10^11^ CFUmL^-1^, the lowest lactic acid accumulated of 4.82 gL^-1^ and the lowest glucose concentration remained (9.28 gL^-1^) compared to the control experiment (no resin) and other resins loading concentrations (Table 3). There were significant differences (*P* < 0.05) in the maximum viable cell concentration and lactic acid accumulated at 10 gL^-1^ of Amberlite IRA 67 resin compared to the control culture and cultures with other resins loading.

**Table 3:** Effect of different IRA 67 loading concentrations on the performance of *P. acidilactici* in batch fermentation.

IRA 67 resin loading (g/L)	Maximum viable cell concentration (cfu mL^-1^)	Lactic acid accumulated (gL^-1^)	Glucose concentration (gL^-1^)
0	1.2 × 10^10^± 0.04^d^	8.09 ± 0.07^a^	12.03 ± 0.12^a^
5	1.0 × 10^11^± 0.10^c^	5.18 ± 0.12^b^	10.72 ± 0.08^b^
10	1.3 × 10^11^± 0.06^a^	4.82 ± 0.04^c^	9.28 ± 0.08^c^
15	1.1 × 10^11^± 0.11^b^	5.07 ± 0.09^b^	9.75 ± 0.06^b^
20	1.1 × 10^11^± 0.08^b^	5.06 ± 0.14^b^	9.82 ± 0.12^c^
30	1.0 × 10^11^± 0.12^c^	5.24 ± 0.08^b^	9.80 ± 0.07^c^
40	1.1 × 10^10^± 0.06^e^	5.12 ± 0.09^b^	9.64 ± 0.08^c^


*The results of maximum viable cell concentration and concentration of lactic acid accumulated are the average of triplicate experiments. a, b, c, d, and e: Mean values with the different letters are significantly different. Statistically significant coefficient (*P* < 0.05) are expressed as means ± SE.*

An increase in resin loading concentrations was found to reduce the growth of *P. acidilactici.* At the highest loading concentration of 40 gL^-1^, the maximum viable cell concentration was drastically reduced to 1.1 × 10^10^ CFUmL^-1^ below the control culture without the addition of resin. This might be due to the less amount of glucose available in the culture for cell to grow since glucose was being adsorbed on the anion exchange resin sites. This is based on the observation that the concentration of glucose which remained in the culture with 40 gL^-1^ of resin loading (9.64 gL^-1^) was slightly lower than the cultures grown with resins at loading concentrations of 15–30 gL^-1^ but they demonstrated higher viable rates (10^11^ CFUmL^-1^). The findings from this study are similar to the findings of Dethe et al. (2006) who reported that glucose from the media competes with lactic acid for adsorption and reduces the amount of lactic acid adsorbed on the anion exchange resin sites and consequently decreased the growth yield. The presence of this kind of competitive adsorption may hence reduce the adsorption capacity of Amberlite IRA 67 resin for lactic acid. Garret et al. (2015) verified that the presence of other anion components in the culture did compete with lactic acid for adsorption on anion exchange resin sites and thus blocking the available sites for lactic acid adsorption. The low cell viability in the culture with 40 gL^-1^ resin might also be due to the shear stress induced by such a high concentration of resin. This hypothesis is based on the concentration of lactic acid accumulation, 5.12 gL^-1^ which was similar to the cultures grown with the resins at loading concentrations of 15–30 gL^-1^ but they demonstrated higher viable rates (10^11^ CFUmL^-1^).

### Effect of Amberlite IRA 67 Resin at Different Agitation Speed on the Stability of the Resin and Cultivation Performance of *P. acidilactici* Using 2 L Stirred Tank Bioreactor

**FIGURE 4:**
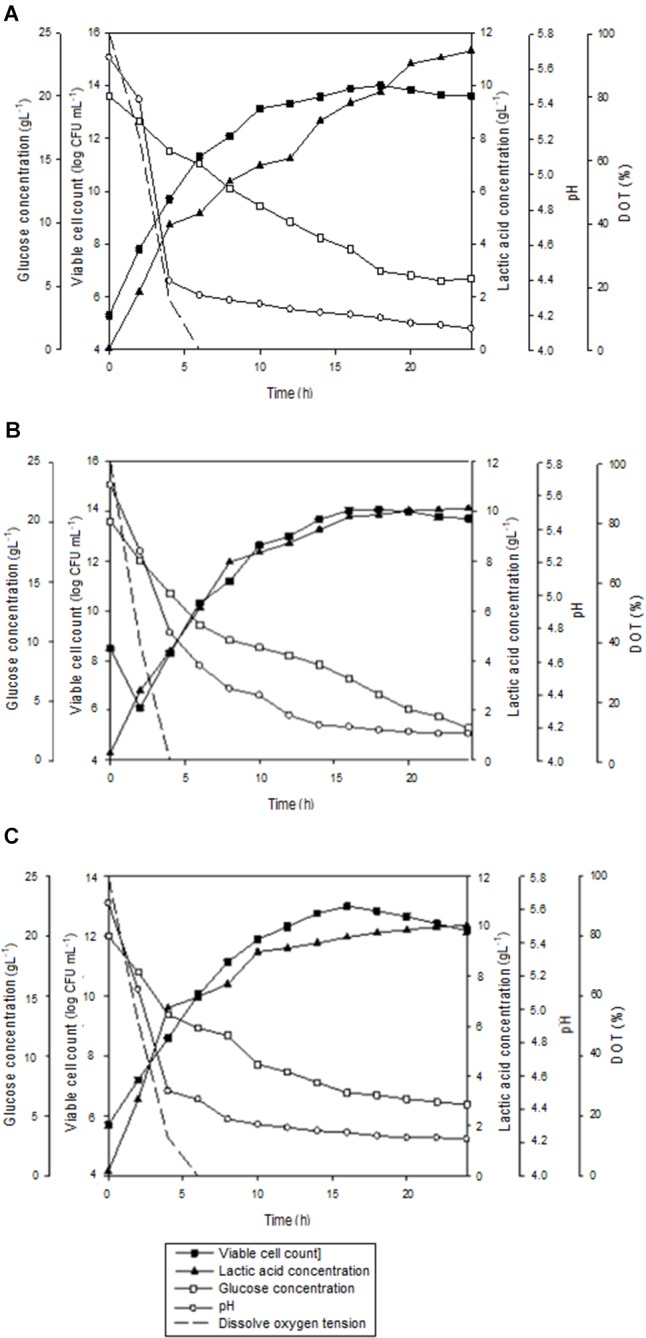
The time course of batch fermentation of *P. acidilactici* in 2 L stirred tank bioreactor with *in situ* addition of resin at 10 gL^-1^ IRA 67 resin at **(A)** 200 rpm **(B)** 300 rpm and **(C)** 400 rpm. The error bar represents the standard deviation about the mean (*n* = 3).

The growth profiles for batch fermentation of *P. acidilactici* in 2 L STR with *in situ* addition of 10 gL^-1^ Amberlite IRA 67 at different agitation speeds are shown in Figure 4. As summarized in Table 4, an increase in agitation speed from 200 to 300 rpm showed an improvement in the maximum viable cell concentration, viable cell yield, viable cell productivity, total lactic acid produced, lactic acid yield and lactic acid productivity. However, as the agitation speed was increased from 300 to 400 rpm, the cultivation performance of *P. acidilactici* was reduced. Of all the three agitation speeds studied, 300 rpm showed the highest viable cell concentration (1.2 × 10^14^ CFUmL^-1^) with improvement of 1.2 and 9.2 times compared to 200 rpm (1.0 × 10^14^ CFUmL^-1^) and 400 rpm (1.3 × 10^13^ CFUmL^-1^), respectively. It was observed that the time taken for the culture to reach maximum viable cell concentration was 2 h longer at both agitation speeds of 200 rpm and 300 rpm compared to the culture at 400 rpm.

**Table 4:** Effect of resin addition at different agitation speed on growth of *P. acidilactici* in batch fermentation using 2 L stirred tank bioreactor.

Kinetic parameter	Agitation speed (rpm)		
	
	200	300	400
Maximum viable cell concentration (cfu mL^-1^)	1.0 × 10^14^± 0.11^b^	1.2 × 10^14^± 0.08^a^	1.3 × 10^13^± 0.06^c^
Time to reach maximum viable cell concentration (h)	18^a^	18^a^	16^b^
Viable cell yield (cfu g_Glucose_^-1^)	8.1 × 10^15^± 0.12^b^	8.3 × 10^15^± 0.07^a^	9.9 × 10^14^± 0.12^c^
Viable cell productivity (cfu (mL.h)^-1^)	5.6 × 10^12^± 0.07^b^	6.7 × 10^12^± 0.12^a^	8.1 × 10^11^± 0.07^c^
Lactic acid accumulated (gL^-1^)	11.31 ± 0.04^a^	10.12 ± 0.09^b^	10.04 ± 0.11^b^
Total lactic acid produced (gL^-1^)	12.97 ± 0.07^b^	14.32 ± 0.05^a^	12.89 ± 0.09^b^
Lactic acid yield (gg_Glucose_^-1^)	1.11 ± 0.04^a^	1.23 ± 0.07^a^	1.10 ± 0.04^a^
Lactic acid productivity (g(L.h)^-1^)	0.54 ± 0.06^a^	0.59 ± 0.05^a^	0.53 ± 0.04^a^


*The results presented are the average of triplicate experiments. a, b, and c: Mean values with the different letters are significantly different. Statistically significant coefficient (*P* < 0.05) are expressed as means ± SE.*

Glucose consumption by *P. acidilactici* was found to be increased from 200 rpm (71.9%) to 300 rpm (86.7%) and then reduced at 400 rpm (70.2%). The highest viable cell yield was obtained at 300 rpm (8.3 × 10^15^ CFUg_Glucose_^-1^), followed by 200 rpm (8.1 × 10^15^ CFUg_Glucose_^-1^) and 400 rpm (9.9 × 10^14^ CFUg_Glucose_^-1^). This trend was similar to the viable cell productivity where 300 rpm showed the highest viable cell productivity [6.7 × 10^12^ CFU(mL.h)^-1^] compared to 200 rpm [5.6 × 10^12^ CFU(mL.h)^-1^] and 400 rpm [8.1 × 10^11^ CFU(mL.h)^-1^]. The results indicated that although the maximum viable cell concentration were reached 2 h earlier in the culture stirred at 400 rpm, the productivity was only equivalent to the culture grown at the slowest agitation speed of 200 rpm.

The highest final pH in the culture was detected at 400 rpm (pH 4.22) compared to 200 rpm (pH 4.12) and 300 rpm (pH 4.16) due to the lowest accumulation of lactic acid in the culture at 400 rpm (10.04 gL^-1^) compared to 200 rpm (11.31 gL^-1^) and 300 rpm (10.12 gL^-1^). The highest total lactic acid produced from the fermentation of *P. acidilactici* was found at 300 rpm (14.32 gL^-1^) with the highest lactic acid yield (1.11 g g_Glucose_^-1^) and lactic acid productivity (1.23 g(L.h)^-1^) compared to those obtained at 200 rpm and 400 rpm. The over production of lactic acid seemed to be dependent on other carbon sources such as peptone, meat extract and yeast extract besides the supplemented glucose in MRS media. Besides glucose, amino acids and proteins also act as carbon and energy source (Hayek and Ibrahim, 2013). Peptones for example are a complex mixture of peptides that may be assimilated as nitrogen and carbon sources (Philon et al., 1998).

**FIGURE 5:**
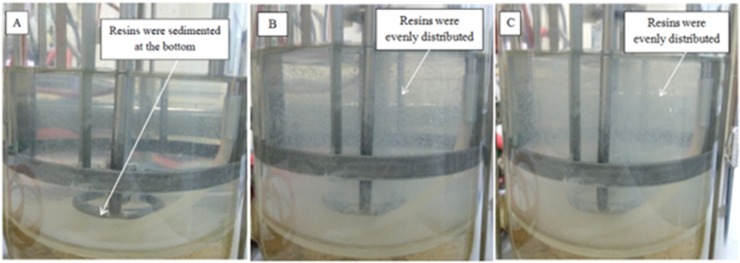
Photographs of dispersed IRA 67 resins in distilled water at agitation speed of **(A)** 200 rpm **(B)** 300 rpm and **(C)** 400 rpm.

Agitation speed is known to be one of the vital factors in designing a resin system (Simsek et al., 2015). Figure 5 shows the flow patterns produced by Rushton turbine in 2 L STR at 200, 300, and 400 rpm with the presence of resin. The resins were horizontally dispersed from the agitator and the flow was distributed to all parts of the vessel with increasing agitation speed. At agitation speed of 200 rpm (Figure 5A), the resins were not evenly distributed, instead most resins were sedimented at the bottom of the vessel. This could be the reason for the significantly higher (*P* < 0.05) lactic acid accumulated in the culture at 200 rpm compared to 300 rpm and 400 rpm although the total lactic acid produced was almost similar to the culture at 400 rpm and lower than the culture at 300 rpm. In other words, uneven dispersal of resins throughout the liquid culture at low agitation speed reduced the adsorption of lactic acid on resin due to less contact between resin and lactic acid.

**FIGURE 6:**
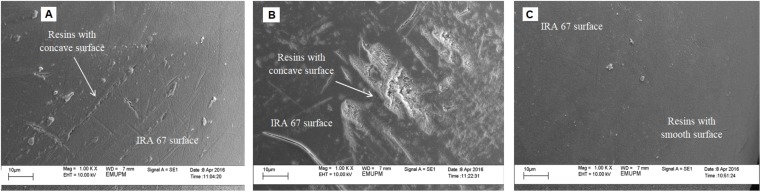
Scanning electron photographs (magnification at ×1,000) of surface structures of IRA 67 resins in **(A)** dispersed condition with agitation speed of 300 rpm and **(B)** 400 rpm and **(C)** in integrated bioreactor-internal column at agitation speed of 300 rpm.

Apparently, resins with a concave surface were detected at agitation speed of 300 rpm (Figure 6A) and 400 rpm (Figure 6B). This observation is similar to the study conducted by Tan et al. (2013) where resins with a concave surface were observed in the fermentation of *E. coli* for *in situ* removal of acetate using anion-exchange resin (Diaion WA30) to enhance the production of periplasmic interferon alpha-2b. They also found that the *in situ* addition of dispersed resin in the culture created shear stress by resin collisions and caused direct shear force to the cells. A possible reason for the low lactic acid eluted from resins (2.85 gL^-1^) in the culture at 400 rpm compared to 300 rpm (4.2 gL^-1^) may be due to the resin capacity to adsorb lactic acid on its site was reduced due to the concave surface of resin caused by higher shear stress created by higher rate of resins collision. It is also tempting to speculate that the decrease in the maximum viable cell concentration of *P. acidilactici* at 400 rpm could be due to the direct shear force to the cells caused by resins collision.

Agitation speed of 300 rpm with *in situ* addition of resin provided the most suitable degree of mixing for the growth of *P. acidilactici*. As shown in Table 5, the growth of *P. acidilactici* with *in situ* resin addition was found to be improved by 67 times (1.2 × 10^14^ CFUmL^-1^) compared to the fermentation without resin addition (1.8 × 10^12^ CFUmL^-1^). However, the time taken for the culture to reach maximum viable cell concentration was 6 h longer than the control culture (without resin addition). Glucose consumption by *P. acidilactici* was found to be increased by 1.3 times in the fermentation with resin addition (17.33 gL^-1^) compared to the control fermentation without resin addition (13.68 gL^-1^). In the meantime, lactic acid accumulation in the culture was lower in the fermentation with resin addition (10.12 gL^-1^) than the control fermentation without resin addition (13.21 gL^-1^). Consequently, the final pH of the culture with resin addition was higher (pH 4.16) than the control fermentation without resin addition (pH 3.95).

**Table 5:** Comparison for cultivation with and without the addition of resin under different bioreactor systems for lactic acid removal on growth of *P. acidilactici* in batch fermentation using 2 L stirred tank bioreactor.

Kinetic parameter	With resin addition	Without resin addition	Resin with column
Maximum viable cell concentration (cfu mL^-1^)	1.2 × 10^14^ ± 0.08^a^	1.8 × 10^12^ ± 0.12^b^	1.7 × 10^14^± 0.07^a^
Time to reach maximum viable cell concentration (h)	18^a^	12^b^	16^b^
Viable cell yield (cfu g_Glucose_^-1^)	8.3 × 10^15^ ± 0.07^a^	2.0 × 10^14^ ± 0.1^b^	9.2 × 10^15^± 0.05^a^
Viable cell productivity [cfu (mL.h)^-1^]	6.7 × 10^12^ ± 0.12^a^	1.5 × 10^11^ ± 0.15^b^	7.8 × 10^12^± 0.08^a^
Lactic acid accumulated (gL^-1^)	10.12 ± 0.09^b^	13.21 ± 0.14^a^	10.05 ± 0.06^a^
Lactic acid produced (gL^-1^)	14.32 ± 0.05^a^	13.21 ± 0.14^b^	14.89 ± 0.08^a^
Lactic acid yield (gg_Glucose_^-1^)	1.23 ± 0.07^a^	0.92 ± 0.08^b^	1.23 ± 0.07^a^
Lactic acid productivity [g(L.h)^-1^]	0.59 ± 0.05^a^	0.54 ± 0.08^b^	0.61 ± 0.04^a^


*The results presented are the average of triplicate experiments. a, b, and c: Mean values with the different letters are significantly different. Statistically significant coefficient (*P* < 0.05) are expressed as means ± SE.*

The results of this study clearly shown that the *in situ* addition of anion-exchange resin in the fermentation of *P. acidilactici* enhanced the growth by reducing the inhibitory effect of lactic acid. The cultures with lactic acid removal system have a lower osmotic pressure than the cultures without lactic acid removal system and therefore, the inhibition of cell growth due to osmotic pressure could also be reduced (Cui et al., 2016).

Among all the agitation speeds studied for *in situ* addition of dispersed resin, the agitation speed of 300 rpm showed the most suitable degree of mixing for growth of *P. acidilactici* and also provided fit environment conditions, such as optimum pH for growth due to low lactic acid level in the culture. Therefore, agitation speed of 300 rpm was selected and used for the subsequent experiments.

### Integrated Bioreactor-Internal Column System for the Removal of Lactate Using Amberlite IRA 67 to Enhance Cultivation Performance of *P. acidilactici*

*In situ* lactic acid removal using Amberlite IRA 67 with an integrated internal column was conducted to study the feasibility of the system to overcome the problem of shear stress created by resin collisions and to further enhance the cultivation performance of *P. acidilactici*.

**FIGURE 7:**
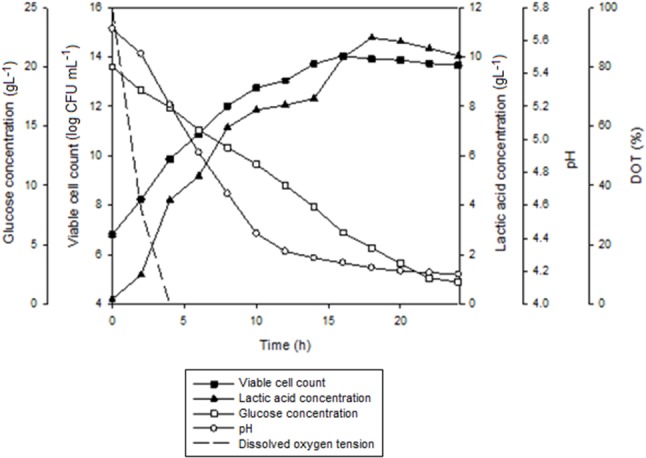
The time course of batch fermentation of *P. acidilactici* in 2 L stirred tank bioreactor with *in situ* addition of 10 gL^-1^ IRA 67 resin using an internal column. The fermentation was conducted at 300 rpm. The error bar represents the standard deviation about the mean (*n* = 3).

Figure 7 shows the time course of batch fermentation of *P. acidilactici* in 2 L STR with *in situ* resin addition using an internal column. Based on Table 5, maximum viable cell concentration in the culture with resin using an internal column (1.7 × 10^14^ CFUmL^-1^) was 1.4 times higher than that obtained in the culture with dispersed resin (1.2 × 10^14^ CFUmL^-1^). This was due to the reduction in the influence of the direct shear force to the cells from the shear stress caused by the resins collision.

Furthermore, the time taken for the culture to achieve maximum viable cell concentration with internal column resin was 2 h shorter than the culture with dispersed resin. The effect probably due to the slightly efficient glucose consumption in the culture with internal column resin (90.6%) compared to the culture with dispersed resin (86.7%). Viable cell yield and viable cell productivity obtained in the culture with internal column resin were 1.1 times and 1.2 times, respectively, higher than that obtained in the culture with dispersed resin.

Total lactic acid produced in the culture with internal column resin (14.89 gL^-1^) was only a slightly higher than that obtained in the culture with dispersed resin (14.32 gL^-1^). Lactic acid yield (1.23 g g_Glucose_^-1^) for both resin systems was equivalent while lactic acid productivity was slightly higher in the culture with internal column resin [0.61 g(L.h)^-1^] as compared to the culture with dispersed resin [0.59 g(L.h) ^-1^].

Resins with concave surface were observed in the culture with dispersed resin conditions (Figure 6A), whereas, resins with smooth surface were observed in the culture using internal column (Figure 6C), indicating that the usage of a column reduced the shear force caused by resin collisions. The improvement of cultivation performance of *P. acidilactici* in the culture with internal column resin in comparison to that of the culture with dispersed resin was achieved due to the suitable environment conditions created by this method, such as low shear effect and shear force caused by resins collision at high agitation speed, low lactic acid level in the culture and suitable pH for improved growth of the strain. The application of resin using column has also been applied to overcome product inhibition and to simplify recovery process of lactic acid, such as lactic acid recovery from cassava bagasse based fermented medium using anion exchange resin (John et al., 2008), high density culture of *L. plantarum* (Cui et al., 2016), improved production of periplasmic interferon alpha-2b by *E. coli* using anion-exchange resin for *in situ* removal of acetic acid in the culture (Tan et al., 2013) and purification of lactic acid obtained from a fermentative process of cassava syrup using ion exchange resins (Quintero et al., 2012). Furthermore, the useful life or operating cycles of IRA-67 used in dispersed phase is expected to be significantly reduced when compared to resin used with internal column as the concave surface may increase the fouling in resin surface clogging the resin pores (Ferrer-Polonio et al., 2016).

## Conclusion

The results of this study indicated that the *in situ* addition of selected anion exchange resin into the fermentation culture was able to reduce the inhibitory effect of lactic acid on the growth of *P. acidilactici.* Among the five anion exchange resins tested, Amberlite IRA 67 at loading concentration of 10 g/L gave the highest removal of lactic acid from the culture, which in turn, improved the growth of *P. acidilactici*. Extractive fermentation employing dispersed resins with agitation speed of 300 rpm showed 1.2 and 9.2 times improvements compared to 200 rpm and 400 rpm, respectively. Meanwhile, the application Amberlite IRA 67 using an internal column in STR resulted in 1.4 and 94 times of improvement in growth of *P. acidilactici* compared to the batch fermentation with dispersed resin and without resin, respectively. The system managed to overcome the problem of shear force exerted on the cells that was created by resins collision as observed in dispersed condition. Anion-exchange resins are reusable and they can be reused after regeneration with recommended regenerant. Therefore, the application of anion-exchange resin for *in situ* removal of lactic acid is highly efficient and cost economical to be used in industry.

## Author Contributions

MH, AA, and MO planned the experiments. MO did the experiments and analysis. MK did the analysis. MO and MH wrote the manuscript. MH, AA, and LR-S revised the manuscript.

## Conflict of Interest Statement

The authors declare that the research was conducted in the absence of any commercial or financial relationships that could be construed as a potential conflict of interest.

## Abbreviations

CFU: colony forming unit
LAB: lactic acid bacteria
MRS: De Man Rogosa and Sharpe
NaOH: sodium hydroxide
RP-HPLC: reverse-phase high performance liquid chromatography
Rpm: rotation per minute
vv^-1^: volume per volume
wv^-1^: weight per volume
